# Soil Saprobic Fungi Differ in Their Response to Gradually and Abruptly Delivered Copper

**DOI:** 10.3389/fmicb.2020.01195

**Published:** 2020-06-17

**Authors:** Polina Golubeva, Masahiro Ryo, Ludo A. H. Muller, Max-Bernhard Ballhausen, Anika Lehmann, Moisés A. Sosa-Hernández, Matthias C. Rillig

**Affiliations:** ^1^Institut für Biologie, Freie Universität Berlin, Berlin, Germany; ^2^Berlin-Brandenburg Institute of Advanced Biodiversity Research (BBIB), Berlin, Germany

**Keywords:** gradual and abrupt stress, heavy metal stress, temporal dynamics, filamentous fungi, copper toxicity, environmental change

## Abstract

The overwhelming majority of studies examining environmental change deliver treatments abruptly, although, in fact, many important changes are gradual. One example of a gradually increasing environmental stressor is heavy metal contamination. Essential heavy metals, such as copper, play an important role within cells of living organisms but are toxic at higher concentrations. In our study, we focus on the effects of copper pollution on filamentous soil fungi, key players in terrestrial ecosystem functioning. We hypothesize that fungi exposed to gradually increasing copper concentrations have higher chances for physiological acclimation and will maintain biomass production and accumulate less copper, compared to fungi abruptly exposed to the highest copper concentration. To test this hypothesis, we conducted an experiment with 17 fungal isolates exposed to gradual and abrupt copper addition. Contrary to our hypothesis, we find diverse idiosyncratic responses, such that for many fungi gradually increasing copper concentrations have more severe effects (stronger growth inhibition and higher copper accumulation) than an abrupt increase. While a number of environmental change studies have accumulated evidence based on the magnitude of changes, the results of our study imply that the rate of change can be an important factor to consider in future studies in ecology, environmental science, and environmental management.

## Introduction

Most ecosystems are subjected to anthropogenic and natural environmental drivers that can represent stressful changes for biota. The effects of anthropogenic stressors have been largely studied by investigating responses to different magnitudes of stress. However, other temporal characteristics of stress may be equally important (Ryo et al., 2019). For instance, experiments typically focus solely on the intensity of change, often without taking the rate of change into account. Different rates of environmental change can have diverging impacts on ecological systems (Klironomos et al., 2005; Siteur et al., 2016), yet the potential importance of the rate of change remains largely unexplored.

Repeated or continuous contamination of a site can lead to a gradual increase in the contaminant concentration in ecosystems over time (Komárek et al., 2010; Ballabio et al., 2018). Soil and groundwater contamination is currently one of the greatest concerns related to soil resources in Europe and across the globe (Tóth et al., 2016). Heavy metal contamination is the most common type of soil and groundwater contamination in Europe (35% and 32%, respectively, of contamination cases in Europe) (Science Communication Unit and University of the West of England, 2013). For example, copper has been used extensively as a fungicide in agriculture, the wood industry, and other fields of human activity for more than three centuries (Karunasekera, 2017). In addition, this type of contamination is practically irreversible, because unlike organic compounds, heavy metals cannot be degraded, only potentially immobilized or extracted by hyperaccumulating organisms (e.g., plants and fungi).

Copper plays an important role within the cells of living organisms – it is a cofactor of proteins, a component of metalloenzymes (Nevitt et al., 2012) and is needed for homeostatic maintenance. However, a tightly coordinated orchestration of uptake, distribution, and efflux in cells is needed (Prohaska, 2008) to prevent toxic effects at higher concentrations. An excess of copper ions can cause fatal cell damage (Eaton and Hale, 1993; Karunasekera, 2017) due to its binding to functional groups, replacing cations, inducing oxidative stress (Zhang et al., 2015), and affecting the membrane transport system (Cervantes and Gutierrez-Corona, 1994; Karunasekera, 2017). These changes inside of fungal hyphae, induced by an excess of copper, can lead to reduced fungal biomass production. Given the abovementioned physiological mechanisms, the rate of copper concentration increase in the environment must be critical for fungal growth and copper uptake. Nonetheless, so far, the overwhelming majority of experimental studies on the effects of environmental change use treatments that are delivered abruptly, although in fact, many important changes in the environment are gradual in nature (Gomaa and Azab, 2013).

In our study, we selected a set of 17 filamentous fungal strains comprising three fungal phyla (Ascomycota, Basidiomycota, and Mucoromycota) which were isolated from the same soil. These fungi are abundant in their ecosystem, are culturable, and show high versatility in trait expression (Andrade-Linares et al., 2016; Lehmann et al., 2019). We focus on the effects of copper on filamentous soil fungi, key players in terrestrial ecosystems functioning (Went and Stark, 1968) by virtue of their role in biogeochemical cycling and immobilization of toxicants (Brookes, 1995; Giller et al., 1998).

With this set of fungi, we here test the hypothesis that fungi exposed to a gradual increase in copper concentration will have a greater chance for physiological acclimation to copper stress and will show higher biomass production and lower copper accumulation. For this, we investigate in an experiment how the rate of copper application (gradual vs. abrupt) affects fungal biomass and copper accumulation. In a second step, we wish to investigate with these data, (1) if fast-growing strains are more at risk to experience biomass reduction compared to slow-growing strains and if this effect is more pronounced under abrupt copper application; (2) we investigate the impact of the rate of copper application on the trade-off between biomass production and copper accumulation. This relationship reflects stress-response strategies of fungi, which can differ depending on the individual properties of the isolates and type of treatment.

## Materials and Methods

### Fungal Isolates and Experimental Design

We used 17 fungal strains originally isolated from a nature protection area of “Oderhänge Mallnow,” a natural, semi-arid grassland in northeast Germany (Mallnow Lebus, Brandenburg 52°27.778′ N, 14°29.349′ E) (Andrade-Linares et al., 2016). Details on the fungal isolates are presented in Supplementary Table S1 in Supplementary Material.

For the experiment, isolates were cultured from −80°C stock cultures on PDA (potato dextrose agar; Sigma-Aldrich, Taufkirchen, Germany) at 20°C (the temperature at which the fungi were originally cultured). Then, one standardized plug (∼0.25 cm^2^) per cup was used to inoculate liquid cultures. The incubations were carried out in a filter-sterilized, chemically defined medium (Czapek-Dox broth, Sigma-Aldrich, Taufkirchen, Germany), adjusted to pH 5.5. The cultures were kept in ventilated incubators at 20°C and were orbitally shaken at 150 rpm. The experimental units consisted of sterile plastic cups (150 ml total volume) filled with the growth medium (50 ml), and there were three treatments (control, gradual, and abrupt), five replicates for each of 17 fungal isolates, summing up to 255 experimental units in total.

### Treatment Application

The liquid cultures of the single fungal isolates received gradual and abrupt copper treatments. The treatments were designed according to a dose-day approach and, thus, the copper addition for the gradual treatment had to be started earlier than for the abrupt one. This way, we kept the duration of incubation the same for both treatments to assure that experimental units received the same overall dose (note that dose-days are calculated as the area under the curve). The final concentration of copper was always the same between abrupt and gradual treatments (see Supplementary Figure S1 in Supplementary Material). The final copper concentration in the growth medium in both treatments was 1 mM of copper sulfate pentahydrate. This concentration caused a significant decrease in biomass production, but was not lethal, according to our preliminary tests.

Fungal cultures were pre-incubated for 5 days without copper addition and then, during the treatment phase (copper addition), incubated for 9 days. In the gradual treatment, copper concentration was increased every 24 h by adding 0.1 ml of copper-nutrient solution. In the abrupt treatment, copper concentration was increased on the fifth day directly to the maximal concentration; on all other days, 0.1 ml of nutrient solution (Czapek-Dox broth) was added. For the control samples, 0.1 ml of medium was added daily to prevent a difference in nutrient supply among treatments.

### Measurements

At the end of the experiment, the fungal biomass was retrieved by filtering through a 3-μm nylon mesh using a diaphragm vacuum pump (Vacuubrand). Next, the samples were carefully transferred to pre-weighed, heat-resistant 1.5-ml centrifuge tubes, dried at 105°C, cooled down in a desiccator, and weighed using an analytical balance; the procedure was repeated until the weight of the samples stabilized. Copper accumulation was measured as the amount of copper (mg) per mycelium dry weight (g) (Vieira and Volesky, 2000). Dry fungal biomass was dissolved in “aqua regia” (a mixture of nitric acid and hydrochloric acid) and then Cu content was measured using ICP-OES (Perkin Elmer Optima 2100DV) based on DIN EN 1346 (Vogel et al., 2015).

### Statistical Analysis

All the statistical analyses were done in R version 3.5.3 (R Core Team, 2018).

We quantified the mean differences in fungal biomass and copper accumulation in mycelium as a function of whether copper was added gradually or abruptly. For each isolate, the unstandardized absolute effect sizes (ESs; i.e., absolute difference in mean between treated group and control) of biomass production and copper accumulation were analyzed independently. Taking into account the heterogeneity in isolate performance under control condition, we also estimated the 95% confidence interval (CI) of the ES for each isolate. For comparing the ESs between abrupt and gradual treatments, we conducted a non-parametric bootstrap resampling (9999 iterations) to estimate the difference in mean values of both treatments while accounting for the variability, and estimated the probability that the difference is explained solely by chance.

Then, we tested if for biomass production differences in ES between abrupt and gradual treatments (i.e., absolute difference between ES abrupt and ES gradual) can be explained solely by the growth rate of fungi in the controls. For this, we conducted correlation tests between these two variables (using Pearson’s method). Moreover, we investigated the correlation of ESs between biomass and copper accumulation under both types of treatment (using the function “cor.test” in R). We tested for a phylogenetic signal of the six response variables (fungal biomass and copper accumulation under the control, gradual and abrupt treatments) based on Blomberg’s *K* statistics (Blomberg et al., 2003). We used the function phyloSignal of the R packages “phylosignal” (Keck et al., 2016). This approach was necessary to evaluate if phylogenetic relatedness could bias our inferences.

The datasets and R scripts are available at the GitHub repository.^1^

## Results

### Biomass Production

The tested isolates showed diverse rather than uniform responses to the applied Cu treatments, suggesting that abrupt environmental changes are not necessarily more harmful than gradual ones.

**FIGURE 1:**
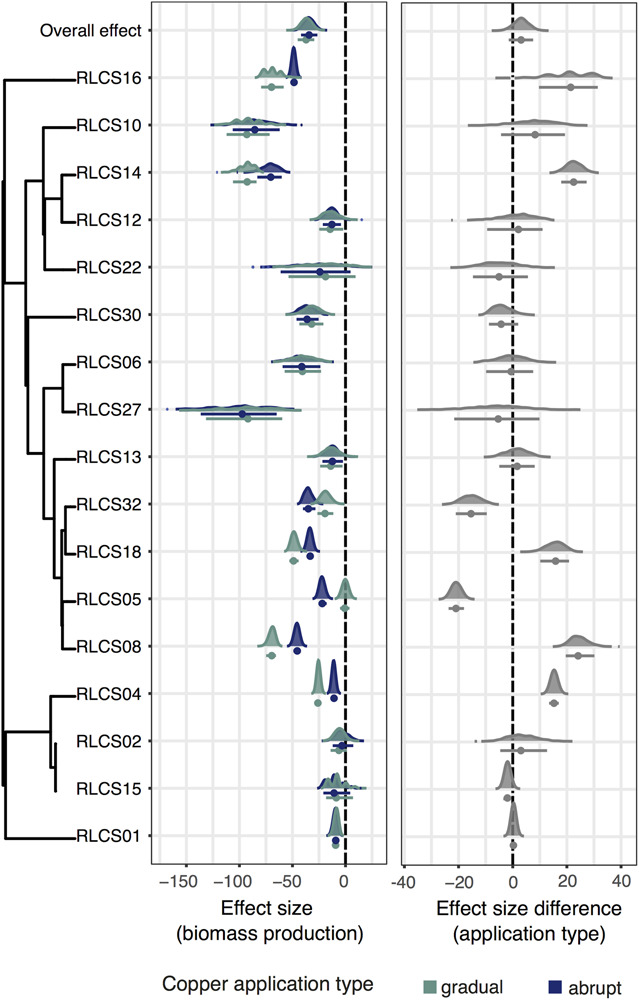
**(Left panel)** Effect sizes of copper treatments on biomass production of each isolate exposed to either gradual or abrupt treatments. **(Right panel)** Difference in the effect sizes. The dots represent means and the bars are 95% confidence intervals. Kernel density plots depict data distributions. The phylogenetic relationship of the fungal isolates is depicted on the left, and we additionally show the overall effect. The vertical dashed lines are the zero effect lines.

We found four different reaction patterns in our isolates (Figure 1). Four isolates (RLCS22, RLCS12, RLCS02, and RLCS13) did not show a significant decrease in biomass production exposed to either treatment. For six other isolates (RLCS10, RLCS15, RLCS27, RLCS06, RLCS30, and RLCS01), biomass was reduced but no difference was detected between the gradual and abrupt treatment. Another five isolates (RLCS04, RLCS14, RLCS18, RLCS16, and RLCS08) had reduced biomass production more strongly under the gradual treatment than in the abrupt treatment (Tukey HSD test, *p* value = 5.16e-08, 1.23e-02, 6.09e-04, 2.39e-02, and 4.57e-05, respectively). In only two cases (RLCS05 and RLCS32) did we find the predicted response, namely, that the abrupt treatment caused more pronounced reduction in biomass production than the gradual treatment (Tukey HSD test, *p* value = 6.31e-06 and 1.11e-02, respectively).

### Copper Accumulation in Fungal Mycelium

**FIGURE 2:**
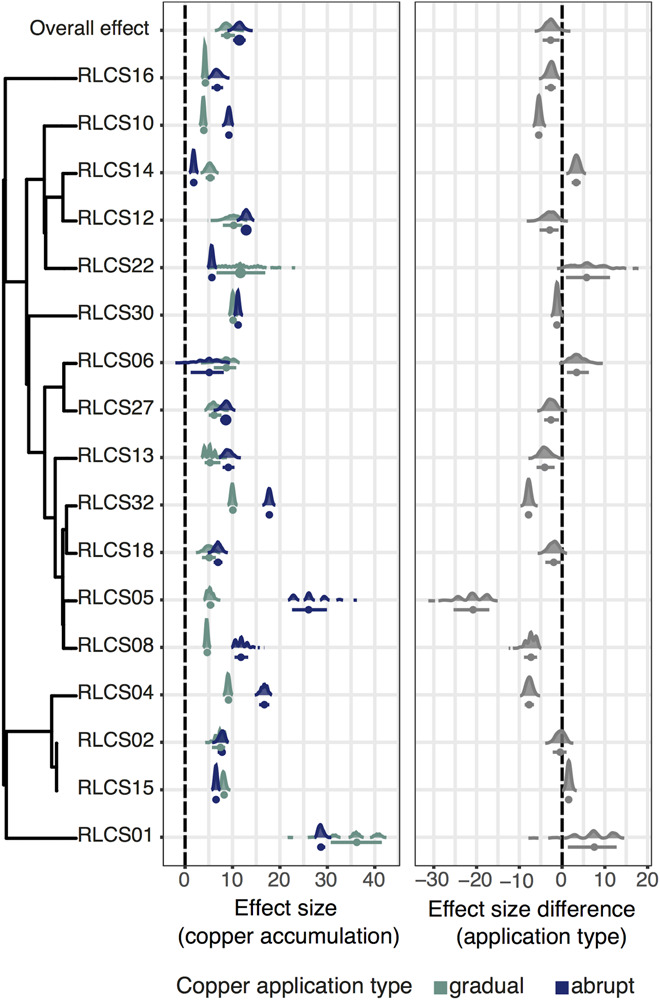
**(Left panel)** Effect of copper treatments on copper accumulation under gradual and abrupt treatments. **(Right panel)** Difference in the effect sizes between the gradual and abrupt treatments. The dots represent means and the bars are 95% confidence intervals. Kernel density plots representing the data distributions. The phylogenetic relationship of the fungal isolates is depicted on the left, and we additionally show the overall effect. The vertical dashed lines are the zero effect lines.

In all cases, when treatments were applied, copper content in fungal biomass was noticeably higher than in the control. When comparing the isolates, contrary to the initial hypothesis, copper accumulation was higher under the gradual treatment for 7 out of the 17 strains (RLCS04, RLCS10, RLCS05, RLCS13, RLCS16, RLCS08, and RLCS32), while only one isolate (RLCS14) accumulated more copper under the abrupt treatment (Figure 2). For the remaining isolates, no difference in copper accumulation between the gradual and abrupt treatments could be detected.

### Relationship Between Copper Effect and Growth Strategy

**FIGURE 3:**
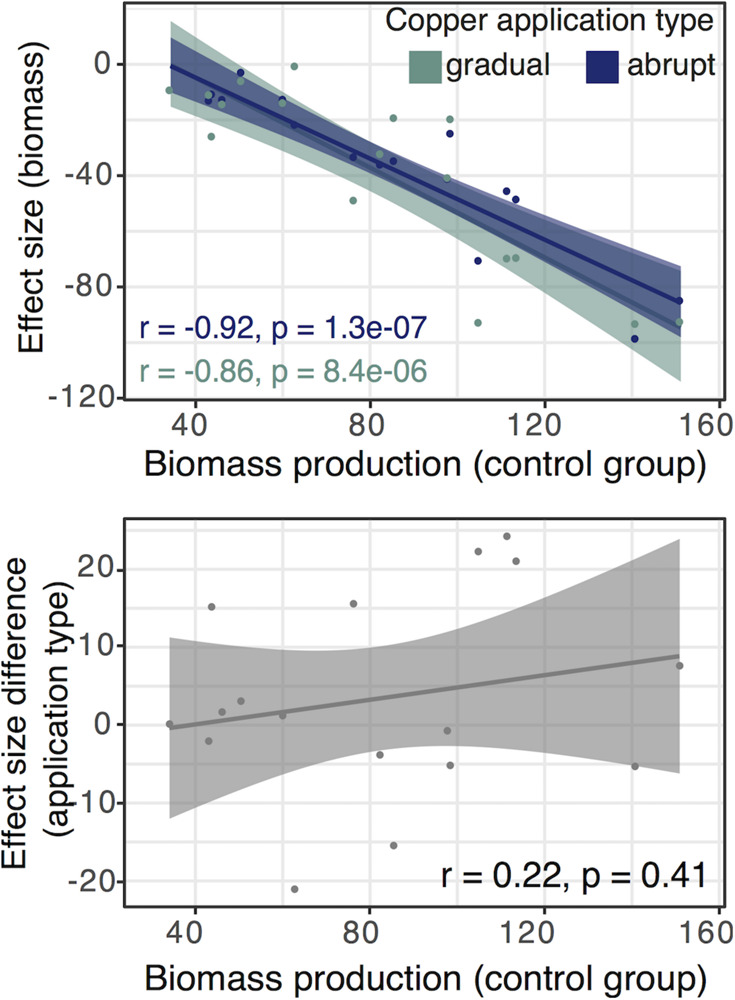
**(Top panel)** Correlation between biomass reduction under the gradual and abrupt treatments and biomass production in the control group. **(Bottom panel)** Correlation between biomass production in the control group and the difference in the effect sizes (ES) between the gradual and abrupt treatments. The difference in ES is not explained by the growth rate of fungi and earlier start of the gradual treatment. ES is calculated as the difference between the treatment and control groups. We show regression lines and confidence intervals for the gradual (green) and abrupt treatment (blue). Each dot represents the mean for one isolate from our set of fungi under the certain treatment.

To test if applied copper treatments were less stressful for fast-growing fungi than slow-growing ones, we investigated the relationship between ES for biomass and the biomass production in the control group. The association was strongly negative in both copper application treatments (ES are negative numbers), indicating an inhibition of biomass production for fast-growing isolates (gradual group: Pearson’s *r* = 0.86, *p* = 8.4e-06, 95% CI [−0.95, −0.65]; abrupt group: Pearson’s *r* = 0.92, *p* = 1.3e-07, 95% CI [−0.97, −0.79]; Figure 3). However, the differences between abrupt and gradual treatments were not explained by the growth rate of fungi (Figure 3).

### Correlation Between Biomass Production and Copper Accumulation

**FIGURE 4:**
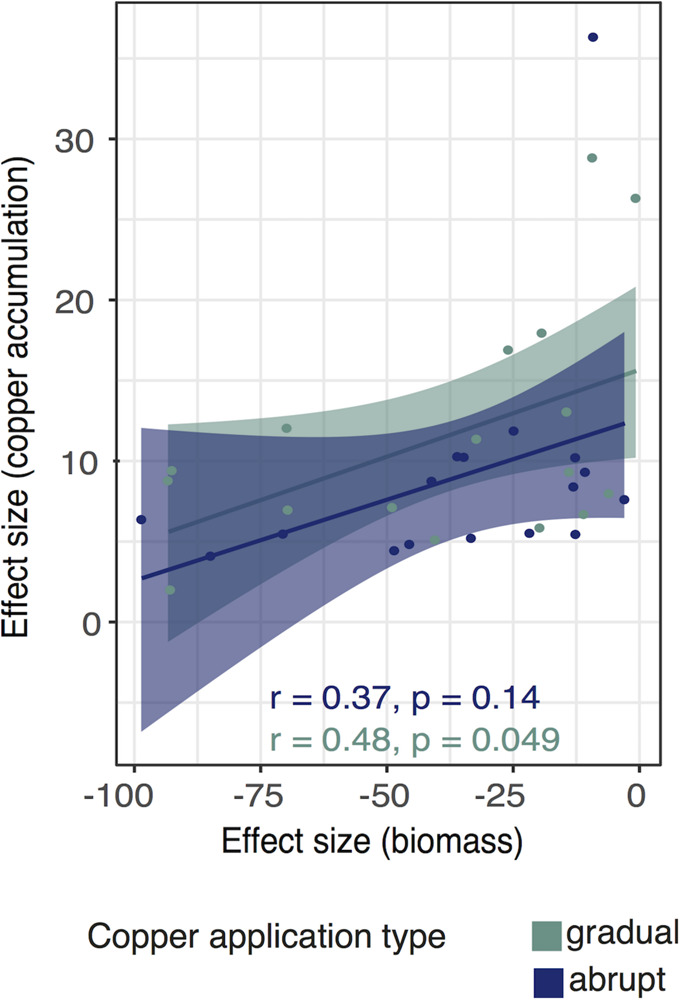
Correlation between biomass reduction and Cu accumulation under the gradual and abrupt treatments. Effect sizes (ES) are calculated as the difference between the treatment and control groups. We show regression lines and confidence intervals for the gradual (green) and abrupt treatment (blue). Dots represent isolate-specific trait mean data for gradual and abrupt treatment applications, respectively.

The correlation between biomass and copper accumulation was positive in both treatments (Figure 4) (gradual group: Pearson’s *r* = 0.49, *p* = 0.05, 95% CI [0.01, 0.78]; abrupt group: Pearson’s *r* = 0.37, *p* = 0.14; 95% CI [−0.13, 0.72]).

### Phylogenetic Signal

Responses were tested for any phylogenetic signal using Blomberg’s *K* statistics. We did not detect any phylogenetic signal (*p* values > 0.05). Hence, we assume that species relatedness did not bias our inferences.

### Additional Observations

Although it was not quantified in our experiment, we observed that, in some cases, mycelium color depended on the treatment type (see Supplementary Figure S2 in Supplementary Material). Two isolates (RLCS05 and RLCS32) had markedly darker mycelium and weaker growth inhibition under the gradual treatment (see Supplementary Figure S2 in Supplementary Material). The RLCS12 isolate had darker mycelium in the abrupt treatment, but the growth was not inhibited by any of the copper treatments (see Supplementary Figure S2 in Supplementary Material).

## Discussion

Contrary to our hypothesis, as well as some other experimental findings (Sivaprakasam et al., 2008), that typically abrupt environmental changes are more harmful than gradual ones, surprisingly, the isolates showed diverse responses to the treatments, rather than responding uniformly. One plausible explanation for the diverse effects of the treatments on biomass production among the isolates could be that a fast-growing fungus (i.e., having a higher biomass production in the control) had already accumulated higher biomass before application of the treatments and then slowed down growth, and therefore the rate of copper addition intrinsically affected these isolates less strongly. Conversely, a slow-grower is more sensitive to the different treatment application rates. Thus, variability of the *effect sizes (biomass)* is related to the growth strategy of the isolates. Nevertheless, the growth rate does not explain the *difference in effect sizes (biomass)* between the gradual and abrupt treatments and did not introduce a bias.

We highlight that the dose-day approach we used here, arguably a reasonable approach to compare different rates of change, inevitably entails a difference in the timing of treatments (see Supplementary Figure S1). This is necessary to achieve equity of the overall dose. This means that the gradual treatment begins to affect fungi earlier during their growth phase. Nonetheless, for the majority of fungal isolates, including fast-growing ones, mycelial growth was noticeably inhibited by both copper treatments; thus, the exact growth phase in which the treatment was started did not affect the outcome here.

Results of the current study also showed that way of copper delivery had a strong effect on copper accumulation by fungal mycelium – it was noticeably higher in the gradual treatment. Possibly, at the early stages of the gradual treatment, fungal cell structures were partly damaged (for example, by copper-induced reactive oxygen species) and when the copper concentration reached the maximum, cells were not able to efficiently apply resource-consuming resistance mechanisms for reducing copper uptake (Prabhakaran et al., 2016). This could explain higher copper accumulation under the gradual copper treatment. Nevertheless, it is important to take into account that accumulation of copper is not an exclusively adaptive and metabolism-dependent process (Turnlund, 1998; Viraraghavan and Srinivasan, 2011). A passive mechanism of accumulation plays an important role and includes binding of copper ions to the cell wall (Viraraghavan and Srinivasan, 2011). This process could also explain higher accumulation of copper under the gradual treatment, which implies longer exposure to stress. The final dose of copper was always the same among the treatments; thus, the difference in accumulation is caused by the difference in stress delivery.

In the current study, we also looked at the correlation between biomass production and copper accumulation. In our opinion, the relationship between *effect size (biomass)* and *effect size (copper accumulation)* represents various response strategies employed by fungi under gradual and abrupt stress. Fungi can have diverse strategies to deal with stress: energy and nutrients can be redirected from mycelial growth to defense and homeostasis maintenance in different ratios. There are a number of “costly” heavy metal defense mechanisms – active efflux of heavy metal ions (Bruins et al., 2000), antioxidant production (Sánchez, 2017; Khan et al., 2018), vacuolar metal compartmentalization (Hall, 2002), metallothionein production (Zhang et al., 2003), and excretion of copper-binding agents (Ross, 1975; Jarosz-Wilkolazka and Gadd, 2003; Anahid et al., 2011). In our study, it was noticed that some fungi (RLCS05, RLCS32, and RLCS12) were producing pigments as protection from heavy metal-induced oxidative stress (Gmoser et al., 2017) and did so differently for the abrupt and gradual treatment. The abovementioned resistance mechanisms can be applied by fungi singly or in various combinations (Bruins et al., 2000; Iram et al., 2009). For example, *Fusarium solani* – a species complex, which includes isolate RLCS12, is able to produce a number of metabolites, including ergosterol, which protects the cells from oxidative stress (Sánchez, 2017; Khan et al., 2018) and thiols, which, when excreted, form complexes with copper ions (Brown and Hall, 1990).

If we assume that there are two ways to use resources – growth and mentioned costly defense mechanisms (for example, an active copper efflux), then we can distinguish four potential strategies or response types of dealing with copper stress: fungi can (a) show extensive growth, but at the same time accumulate high amounts of copper, e.g., invest resources in growth, possibly trying to escape from the stressful environment; (b) show extensive growth and accumulate low amounts of copper, e.g., be naturally more tolerant and being able to invest resources into copper excretion; (c) show strong inhibition of growth and accumulate high amounts of copper, e.g., be naturally more sensitive to copper stress and not being able to continue growth, nor actively defend against copper stress; (d) show strong inhibition of growth, but accumulate low amounts of copper, e.g., invest the resources into defense, not into biomass production.

In the current study, we observed that even though the responses of individual isolates were diverse, the association between *effect size (biomass)* and *effect size (copper accumulation)* was positive in both treatments (Figure 4), meaning that isolates for which biomass was not reduced by treatments tended to accumulate more copper. Although the slope of the regression line does not differ between the treatments, the positive correlation between ES biomass (biomass reduction) and ES copper accumulation was stronger for the gradually than for the abruptly treated one.

Also, according to our observations, fungi employed abovementioned response strategies (a), (b), and (d) and some isolates applied different strategies to deal with abrupt and gradual treatments.

We can conclude that contrary to our hypothesis, conventional wisdom, and previous findings, gradual application of copper overall did not result in better performance in filamentous fungi, and indeed the responses we observed differed widely among the different isolates. Our study was focused on documenting response patterns across a broad suite of fungal isolates. A next step would be to deepen our understanding of the effects of gradual and abrupt copper pollution on filamentous fungi, by elucidating underlying response mechanisms. Some studies report that adaptive strategies can vary at the molecular level (Lindsey et al., 2013; Bleuven and Landry, 2016).

The effect of differences in the rate of change is largely understudied. It is advisable for studies in stress ecology and ecotoxicology to apply a wider range of treatment scenarios (with different rates of change) to fill this important gap. A better understanding of the temporal nature of stress and response is important to predict how environmental changes, including drivers of global change, affect organisms, communities, and functioning of ecosystems (Ryo et al., 2019).

## Data Availability Statement

All datasets generated for this study are included in a github repository: https://github.com/golubevapolina/gradual-vs-abrupt-stress-fungi.

## Author Contributions

PG conducted the study, which was designed by PG, LM, M-BB, and MCR. The manuscript was written through contributions of all authors. All authors have given approval to the final version of the manuscript.

## Conflict of Interest

The authors declare that the research was conducted in the absence of any commercial or financial relationships that could be construed as a potential conflict of interest.

## Footnotes

1 https://github.com/golubevapolina/gradual-vs-abrupt-stress-fungi
