# Supplementary material for: Soil Saprobic Fungi Differ in Their Response to Gradually and Abruptly Delivered Copper
Source: Front Microbiol. 2020 Jun 17;11:1195. doi: 10.3389/fmicb.2020.01195 (PMC7325975; doi:10.3389/fmicb.2020.01195)
Supplement: Supplementary file 1 [file Table_1.DOCX]

**Table S1**. Fungal isolates: Information about phylum, class, order, family, taxon name and Deutsche Sammlung von Mikroorganismen und Zellkulturen (German Collection of Microorganisms and Cell Cultures GmbH, DSMZ) accession numbers of the 17 fungal isolates used in the experiment.

| Strain ID | DSMZ accession number | Phylum | Class | Order | Family | Taxon name* |
| --- | --- | --- | --- | --- | --- | --- |
| RLCS10 | DSM100286 | Ascomycota | Dothideomycetes | Pleosporales | Pleosporaceae | Alternaria alternata |
| RLCS22 | DSM100401 | Ascomycota | Dothideomycetes | Pleosporales | Phaeosphaeriaceae | Paraphoma chrysanthemicola |
| RLCS12 | DSM100405 | Ascomycota | Dothideomycetes | Pleosporales | Didymellaceae | Didymellaceae strain 1 |
| RLCS14 | DSM100404 | Ascomycota | Dothideomycetes | Pleosporales | Didymellaceae | Didymellaceae strain 2 |
| RLCS30 | DSM100291 | Ascomycota | Eurotiomycetes | Chaetothyriales | Herpotrichiellaceae | Exophiala  equina |
| RLCS05 | DSM100403 | Ascomycota | Sordariomycetes | Hypocreales | Nectriaceae | Fusarium sp. |
| RLCS32 | DSM100409 | Ascomycota | Sordariomycetes | Hypocreales | Nectriaceae | Fusarium oxysporum |
| RLCS08 | DSM100325 | Ascomycota | Sordariomycetes | Hypocreales | Nectriaceae | Gibberella tricincta |
| RLCS18 | DSM100287 | Ascomycota | Sordariomycetes | Hypocreales | Nectriaceae | Gibberella sp. |
| RLCS13 | DSM100290 | Ascomycota | Sordariomycetes | Hypocreales | Nectriaceae | Fusarium  solani |
| RLCS27 | DSM100326 | Ascomycota | Sordariomycetes | Sordariales | Chaetomiaceae | Thielavia inaequalis |
| RLCS06 | DSM100400 | Ascomycota | Sordariomycetes | Sordariales | Chaetomiaceae | Chaetomium angustispirale |
| RLCS16 | DSM100408 | Basidiomycota | Agaricomycetes | Agaricales | Pleurotaceae | Pleurotus pulmonarius |
| RLCS15 | DSM100402 | Muceromycota | Mortierellomycetes | Mortierellales | Mortierellaceae | Mortierella elongata strain 1 |
| RLCS02 | DSM100407 | Muceromycota | Mortierellomycetes | Mortierellales | Mortierellaceae | Mortierella elongata strain 2 |
| RLCS04 | DSM100322 | Muceromycota | Mortierellomycetes | Mortierellales | Mortierellaceae | Mortierella  exigua |
| RLCS01 | DSM100293 | Mucoromycota | Mucoromycetes | Mucorales | Mucoraceae | Mucor  fragilis |

*best resolved tree annotation passing 80% threshold of bootstrap approach
