# Supplementary material for: Soil Saprobic Fungi Differ in Their Response to Gradually and Abruptly Delivered Copper
Source: Front Microbiol. 2020 Jun 17;11:1195. doi: 10.3389/fmicb.2020.01195 (PMC7325975; doi:10.3389/fmicb.2020.01195)
Supplement: Supplementary file 2 [file Image_1.PDF]

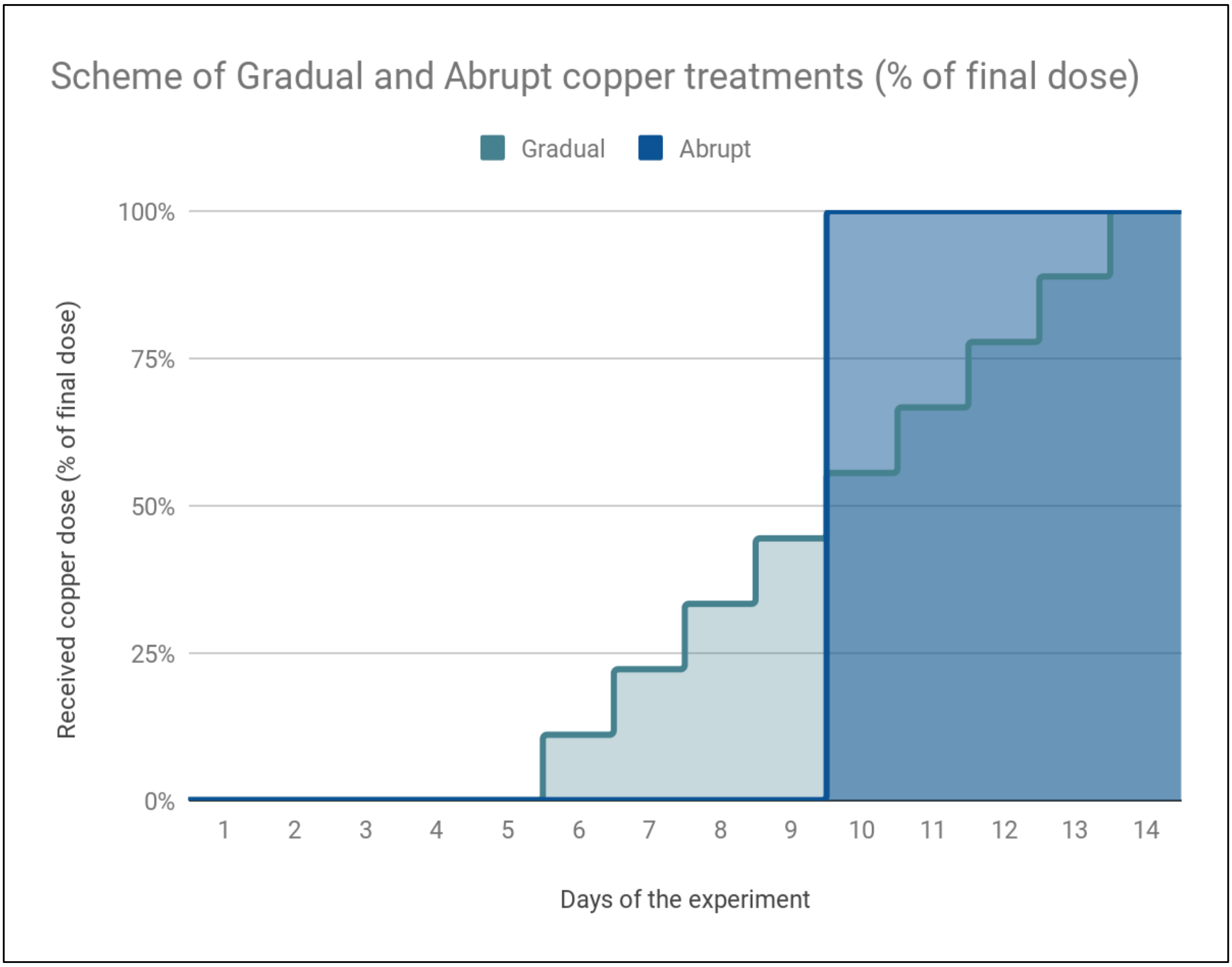

Figure S1. Dose-days calculation. The vertical axis represents copper dose relative to the final dose (unity fraction), and the horizontal axis represents the number of days the fungus spent under the treatment. In case of the gradual treatment (green), copper concentration increases stepwise. In the abrupt treatment (red), copper concentration increases from zero to the maximum, in a single event. The dose-days are shown here as the area under the curve, and it can be calculated as a definite integral of the curve.
