# Supplementary material for: Soil Saprobic Fungi Differ in Their Response to Gradually and Abruptly Delivered Copper
Source: Front Microbiol. 2020 Jun 17;11:1195. doi: 10.3389/fmicb.2020.01195 (PMC7325975; doi:10.3389/fmicb.2020.01195)
Supplement: Supplementary file 3 [file Image_2.PDF]

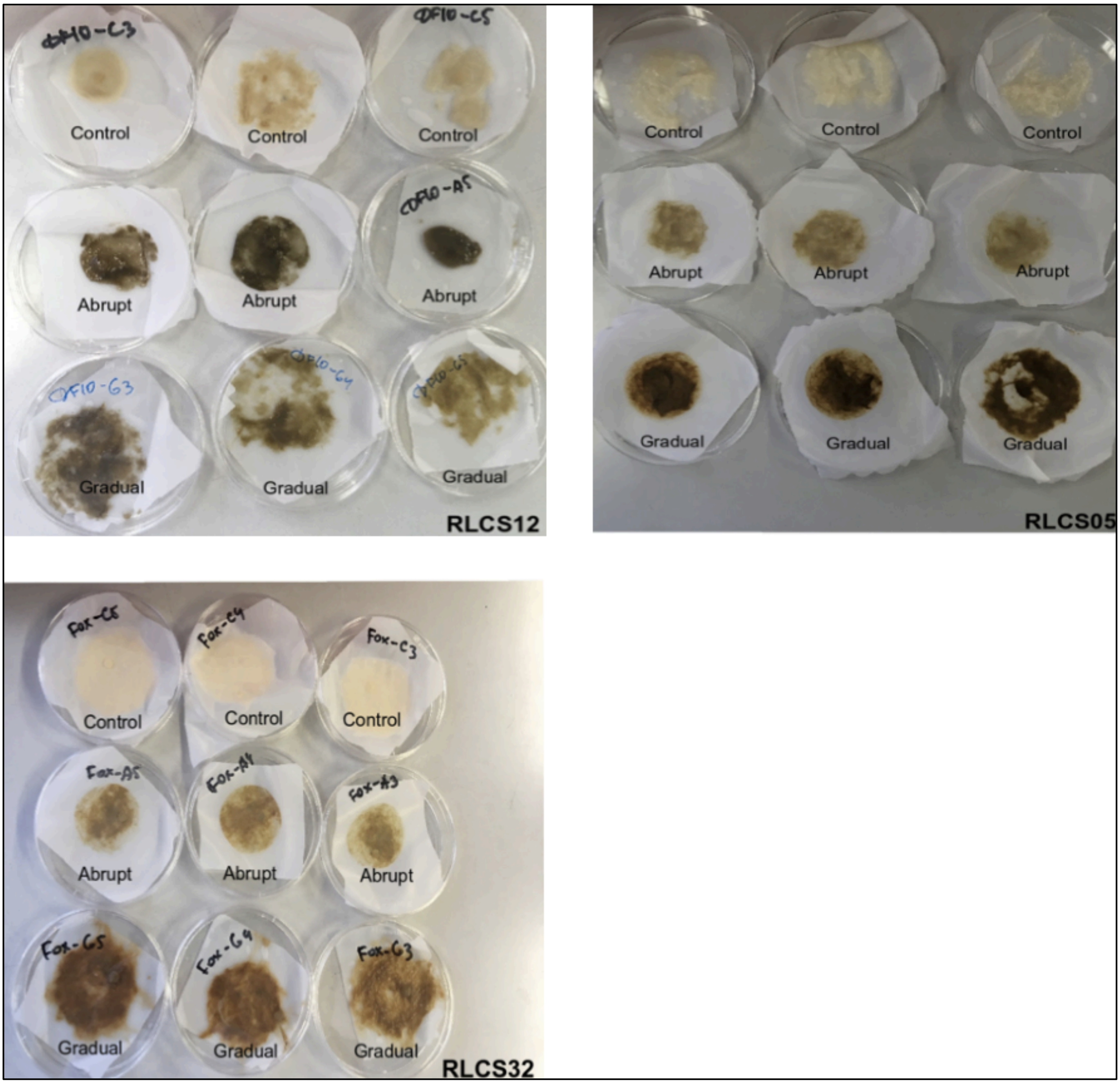

Figure S2. Effect of gradual vs. abrupt copper treatment on the mycelium pigmentation. Mycelium of fungus RLCS12 was darker under abrupt copper stress. Isolates RLCS05 and RLCS32 formed darker mycelium under gradual copper stress.
